# Term amniotic fluid: an unexploited reserve of mesenchymal stromal cells for reprogramming and potential cell therapy applications

**DOI:** 10.1186/s13287-017-0582-6

**Published:** 2017-08-25

**Authors:** Roksana Moraghebi, Agnete Kirkeby, Patricia Chaves, Roger E. Rönn, Ewa Sitnicka, Malin Parmar, Marcus Larsson, Andreas Herbst, Niels-Bjarne Woods

**Affiliations:** 1 Section of Molecular Medicine and Gene Therapy, Lund Stem Cell Center, Lund University, BMC A12, 221 84 Lund, Sweden; 2 Wallenberg Neuroscience Center and Lund Stem Cell Center, Lund University, BMC A11, 221 84 Lund, Sweden; 3 Department of Molecular Hematology, Lund Stem Cell Center, Lund University, BMC B12, 221 84 Lund, Sweden; 4 Skåne University Hospital, Department of Obstetrics, Lund University, Lund, Sweden

**Keywords:** Term amniotic fluid, Caesarean section deliveries, Mesenchymal stromal cells, Cell-based therapy, Cellular reprogramming, Pluripotency, Regenerative medicine, Biobanking

## Abstract

**Background:**

Mesenchymal stromal cells (MSCs) are currently being evaluated in numerous pre-clinical and clinical cell-based therapy studies. Furthermore, there is an increasing interest in exploring alternative uses of these cells in disease modelling, pharmaceutical screening, and regenerative medicine by applying reprogramming technologies. However, the limited availability of MSCs from various sources restricts their use. Term amniotic fluid has been proposed as an alternative source of MSCs. Previously, only low volumes of term fluid and its cellular constituents have been collected, and current knowledge of the MSCs derived from this fluid is limited. In this study, we collected amniotic fluid at term using a novel collection system and evaluated amniotic fluid MSC content and their characteristics, including their feasibility to undergo cellular reprogramming.

**Methods:**

Amniotic fluid was collected at term caesarean section deliveries using a closed catheter-based system. Following fluid processing, amniotic fluid was assessed for cellularity, MSC frequency, in-vitro proliferation, surface phenotype, differentiation, and gene expression characteristics. Cells were also reprogrammed to the pluripotent stem cell state and differentiated towards neural and haematopoietic lineages.

**Results:**

The average volume of term amniotic fluid collected was approximately 0.4 litres per donor, containing an average of 7 million viable mononuclear cells per litre, and a CFU-F content of 15 per 100,000 MNCs. Expanded CFU-F cultures showed similar surface phenotype, differentiation potential, and gene expression characteristics to MSCs isolated from traditional sources, and showed extensive expansion potential and rapid doubling times. Given the high proliferation rates of these neonatal source cells, we assessed them in a reprogramming application, where the derived induced pluripotent stem cells showed multigerm layer lineage differentiation potential.

**Conclusions:**

The potentially large donor base from caesarean section deliveries, the high yield of term amniotic fluid MSCs obtainable, the properties of the MSCs identified, and the suitability of the cells to be reprogrammed into the pluripotent state demonstrated these cells to be a promising and plentiful resource for further evaluation in bio-banking, cell therapy, disease modelling, and regenerative medicine applications.

**Electronic supplementary material:**

The online version of this article (doi:10.1186/s13287-017-0582-6) contains supplementary material, which is available to authorized users.

## Background

Mesenchymal stromal cells (MSCs) are multipotent cells capable of differentiating into various cell types, homing to sites of tissue injury, and secreting bioactive molecules [1]. These cells are immune evasive and possess immunosuppressive functions [2, 3]. Thus, MSCs are an attractive cell population for exploration in cell therapy applications, and are currently being evaluated in numerous pre-clinical and clinical studies for various diseases (reviewed in [1, 4, 5]). MSCs can be extracted from a variety of adult and neonatal sources. However, tissue accessibility, obtaining therapeutic applicable cell doses of MSCs, and differences in in-vivo differentiation potential of MSCs isolated from distinct tissue sources are among the most important considerations for regenerative medicine applications of these cells [6–8]. Invasiveness of the medical procedures for cell extraction from donors and/or extensive laboratory processing of the tissues are additional factors restricting MSC use (reviewed in [8]). Adult MSCs are most commonly extracted from bone marrow (BM) for clinical applications; however, due to their low frequency [9–12], which decreases further with aging [13–15], quantities of harvested cells are generally insufficient for direct clinical application. Long-term in-vitro expansion of these cells is also associated with reduced function and therapeutic potential [16–19]. Thus, to ensure the safety and efficacy of MSC-based clinical trials, the use of freshly isolated or low passage MSCs is recommended. Obtaining MSCs from BM requires invasive medical procedures on donors, adding complication to the ethical justification of this source. Adipose tissue (AD) is an alternative relatively abundant source of adult MSCs, obtainable during required or elective medical procedures. These cells are currently being tested in numerous pre-clinical and clinical applications, but as with all MSC sources, the full functional potential of these cells as specified by their tissue origin, is still unclear. Thus, additional MSC sources are required to be able to provide suitable numbers of the functionally relevant cells. Neonatal tissues such as umbilical cord, placenta, and amniotic membrane are other MSC sources with high proliferation rates and potent differentiation capacities, and thus are being evaluated in pre-clinical models [20–22]. Acquiring MSCs from these neonatal tissues requires invasive medical procedures on donors and/or extensive laboratory processing and their utility has not been evaluated to the extent of BM and adipose MSCs. Low MSC frequency in umbilical cord blood is also problematic. Therefore, having access to alternative abundant and accessible MSC sources could complement or extend uses of MSCs beyond that from current sources. Term human amniotic fluid collection may be an ideal alternative source of MSCs, given their neonatal origin, the number of potential donors of this otherwise discarded material, as well as confirmatory pre-clinical studies in second and third-trimester amniotic fluid cells (reviewed in [23]). However, the current method of acquiring these cells limits the number of cells obtainable per donor. Moreover, only a limited number of studies have characterized the features of the term source of MSCs [24–26]. In this study, we demonstrate the feasibility of collecting significant volumes of term amniotic fluid using a siphoning catheter system, and explore the numbers and features of the term amniotic fluid-derived MSCs (TAF-MSCs). We also evaluate the TAF-MSCs in a reprogramming application for the generation of induced pluripotent stem cells and subsequent differentiation into haematopoietic and neural lineages.

## Methods

### Term amniotic fluid collection, cell isolation, and cultivation procedure

**Fig. 1:**
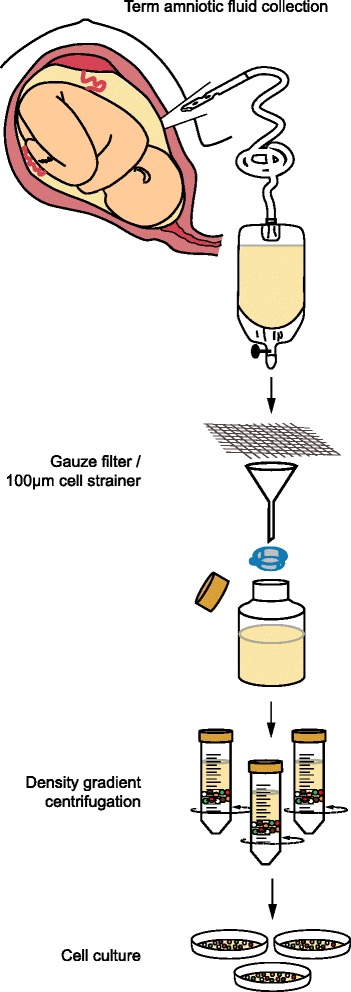
Schematic overview of the procedure used to collect large volumes of term amniotic fluid using a closed catheter-based system, followed by MNC isolation and cell culture

Healthy women undergoing scheduled term caesarean section deliveries (gestational age 37–41 weeks) were informed about the study, and 20 women accepted to participate. Standard operating procedures for caesarean section delivery were performed, but when possible the amniotic membrane was exposed intact. A soft plastic catheter was then used to perforate the amniotic membrane and the tubing was inserted into the amniotic cavity for fluid collection. The catheter system used was a prototype developed for the purpose of high-volume collection of term amniotic fluid. Samples of amniotic fluid were then harvested into an expandable drainage collection vessel through a sealed and sterile tube by passive siphoning. Processing of the cellular material from the fluid was performed within 2–4 hours (Fig. 1). Amniotic fluid was filtered through a sterile mesh gauze pad to remove residual vernix or other large particulates, and then through a 100-μm nylon cell strainer (Fisher Scientific). Filtered samples were centrifuged at 850 × *g* for 5 min and the cell pellet was resuspended in 20–50 ml DMEM + 10% FCS depending on the sample volume. Further separation of mononuclear cells from possible red blood cell contamination was done by density gradient centrifugation of the sample over lymphoprep (Medinor AB or AXIS-SHIELD) at 850 × *g* for 20 min at room temperature. The isolated mononuclear cells were counted by Trypan blue exclusion (Sigma-Aldrich), and evaluated for their clonogenic potential (CFU-F assay) or expansion activity. The MNCs were plated at different plating densities of 1 × 10^4^–6 × 10^4^ cells per cm^2^ on rat tail collagen I (BD Bioscience) pre-coated six-well plates in each of four FCS-based media; medium 1 (EM; ScienCell Research laboratories), medium 2 (FM; ScienCell Research laboratories), medium 3 (StemMACS MSC expansion media; Miltenyi Biotec), and medium 4 (DMEM + 10% FCS). At days 11–14, fibroblastic colony forming units were counted and then individual cell colonies were either picked for clonal cell expansion or were pooled and expanded in all these media. Cells were split every 3 days at a seeding density of 3 × 10^3^–7 × 10^3^ cells per cm^2^ depending on their passage number. Expansion of epithelioid cells was also evaluated in small airway epithelial cell growth medium (SAGM; Lonza).

### Colony forming unit-fibroblast assays

After 11–14 days of culturing cells, the number of colony forming unit-fibroblasts (CFU-F) was scored microscopically by counting the colonies with clear spindle-shaped fibroblast-like morphology and excluding the colonies with round-shape epithelioid-like morphology. Colonies containing ≥ 40 cells were counted.

### Characterization of TAF-derived cells by flow cytometry

Single-cell suspensions from confluent cultures of passages 3–5 in media 1 and 2 were prepared and stained with fluorescent-labelled antibodies. All antibodies were purchased from BD Biosicence. For intracellular staining of OCT4, cells were fixed (4% PFA) and permeabilized (0.5% Triton X-100) before staining. Isotype antibodies served as control. Quantitative analysis was performed using FACSCantoII flow cytometer (BD) and FlowJo software.

### In-vitro differentiation of TAF-derived cells to osteoblasts and adipocytes

Cells expanded in media 1, 2, and 3 for four passages were differentiated towards osteoblastic and adipogenic lineages as described previously [27, 28]. Briefly, cells were cultured in osteoblast induction medium (Miltenyi) for 21 days and stained with Alizarin Red to measure calcium mineral content. For adipogenic differentiation, cells were cultured in AdipDiff medium (Miltenyi) and stained with Oil Red-O to detect lipid vacuoles.

### Gene expression analyses

Affymetrix Gene chip HT 1.1 ST microarrays were used for the analysis. The CEL files were normalized using the RMA method (Robust Multi-array Averaging) using the *oligo* R package from Bioconductor [29, 30]. RMA provides background-adjusted, quantile-normalized and probe-level data summarized values for all probe sets. Differential expression assessment was performed using the limma R package through Bioconductor [30, 31]. The limma package uses linear models to assess differential expression in the context of multifactor designed experiments. For enrichment analyses across published data sets, reported lists of significant genes were overlapped with genes found to be significantly enriched in the present analysis (*p* < 0.05) and subjected to hypergeometric statistics to determine significant of overlap. Venn diagrams were generated online (http://www.cmbi.ru.nl/cdd/biovenn/) [32]. The analysis of present study was performed using technical replicates of both media 1 and 2 cultured cells from a randomly selected donor.

### Haematopoietic differentiation of iPS and hES cell lines

Induced pluripotent stem cells were generated from TAF-MSCs and tested for pluripotency (see Additional file 1 for more details). Next, embryoid bodies (EBs) were formed and differentiated towards haematopoietic cell lineages for 21 days as described previously [33]. Following differentiation, cells were harvested and assessed for their haematopoietic differentiation potential by flow cytometry analysis of CD45, CD43, and CD34 and by colony forming unit assay (CFU assay). Some haematopoietic colonies were stained by May–Grünwald–Giemsa or benzidine to confirm their phenotypes.

### Lymphoid differentiation analyses

After 17 days of hPSC-to-haematopoietic cell differentiation, cells were harvested and sorted for CD43CD34 cell surface expression by FACS. One hundred CD43CD34 double-positive cells were plated on approximately 80% confluent OP9 or OP9-DL1 monolayer stroma cells and differentiated towards lymphoid lineages using an adapted differentiation protocol previously described by Renoux et al. [34] (see Additional file 1 for more details). Following 4 weeks of differentiation, cells were harvested and analysed for T and NK cell surface phenotypes.

### Neural differentiation of iPS cell lines

To assay neural (ectoderm) differentiation potential of the iPS cell lines, a previously described 21-day neural differentiation protocol [35] was used, but modified to remove dual inhibition of SMAD signalling in order to assess the intrinsic differentiation bias of the lines towards neural and non-neural lineages. Briefly, neural induction was stimulated by embryoid body formation, but in the absence of SB431542 and Noggin from the medium; see extended experimental procedure for more details. At day 21, cells were either collected for qRT-PCR analyses or fixed (4% PFA) for immunocytochemistry using antibodies against beta-III-tubulin (TUJ1, rabbit; Covance) and TE7 (mouse; Millipore) (see Additional file 1 for more details).

### Statistical analyses

The data in this study are presented as mean ± SEM unless otherwise stated.

## Results

### Term amniotic fluid collection

The collection procedure of amniotic fluid from healthy mothers undergoing scheduled term caesarean section (gestational week 37–41) is shown in Fig. 1. In total, 20 samples of amniotic fluid were harvested from 19 caesarean sections (two samples were harvested from each amniotic sac in a twin delivery). Aside from these 20 samples, one attempted collection failed because there was almost no amniotic fluid in the amniotic sac (oligohydramnios). The average volume of collected fluid from the 20 samples was 393 ± 81 ml (range 39–1500 ml) (Table 1). The collection added an average of 90 s (range 60–180 s, *n* = 17) to the caesarean section procedure. All newborns were vigorous with Apgar score 9 or 10 at 5 min. Mononuclear cells were isolated from 12 randomly selected samples and the average number of viable MNC obtained was 7.3 ± 2.3 million per litre (range 1–24 million per litre).

**Table 1:** Information associated with caesarean deliveries, newborns, collection of the fluid, as well as fluid volume and cellularity

Sample	Indication for caesarean	Gestational age (week + day)	Duration of collection (s)	Apgar score^a^	AF volume (ml)	Number of MNCs (10^6^/litre)
1	Two previous CSs	38 + 1	70	9, 10, 10	400	10.7
2	Breech presentation	39 + 2	120	7, 9, 10	312	5.5
3	Previous anal sphincter injury	39 + 2	90	10, 10, 10	1025	3.7
4	Previous complicated delivery	39 + 6	ND	9, 10, 10	250	24
5	Two previous CSs	39 + 1	180	9, 10, 10	450	4.8
6	Previous uterine rupture	40 + 1	ND	9, 10, 10	480	22.9
7	Breech presentation	38 + 4	120	9, 10, 10	39	2.8
8	Previous CS + maternal request	38 + 6	60	9, 10, 10	359	1.0
9	Placenta praevia	38 + 3	100	6, 10, 10	219	5.8
10	Transverse lie	37 + 6	70	7, 9, 10	410	2.6
11	Breech presentation	41 + 0	70	9, 10, 10	119	1.8
12	Breech presentation	37 + 0	ND	8, 9, 10	750	ND
13 and 14^b^	Twins + previous placenta accreta	37 + 1	90 + 45	9, 10, 10 + 9, 10, 10	86 + 55	ND
15	Previous CS + maternal request	39 + 0	90	8, 9, 10	1500	ND
16	Two previous CSs	38 + 4	170	9, 10, 10	650	ND
17	Previous CS + maternal request	38 + 6	90	9, 9, 10	250	ND
18	Previous CS + maternal request	39 + 1	120	8, 9, 10	320	ND
19	Placenta praevia	38 + 4	60	9, 10, 10	131	ND
20	Previous CS + maternal request	38 + 3	90	10, 10, 10	55	1.9

*AF* amniotic fluid, *CS* caesarean section, *MNC* mononuclear cell, *ND* not determined

^a^Apgar score represents vitality signs on a scale of 0–10 at 1, 5, and 10 min

^b^Samples 13 and 14 were harvested from a dichorionic diamniotic twin pregnancy

### Isolated cell colonies from term amniotic fluid reveal fibroblastic and epithelioid cell morphologies with differing proliferative activities

**Fig. 2:**
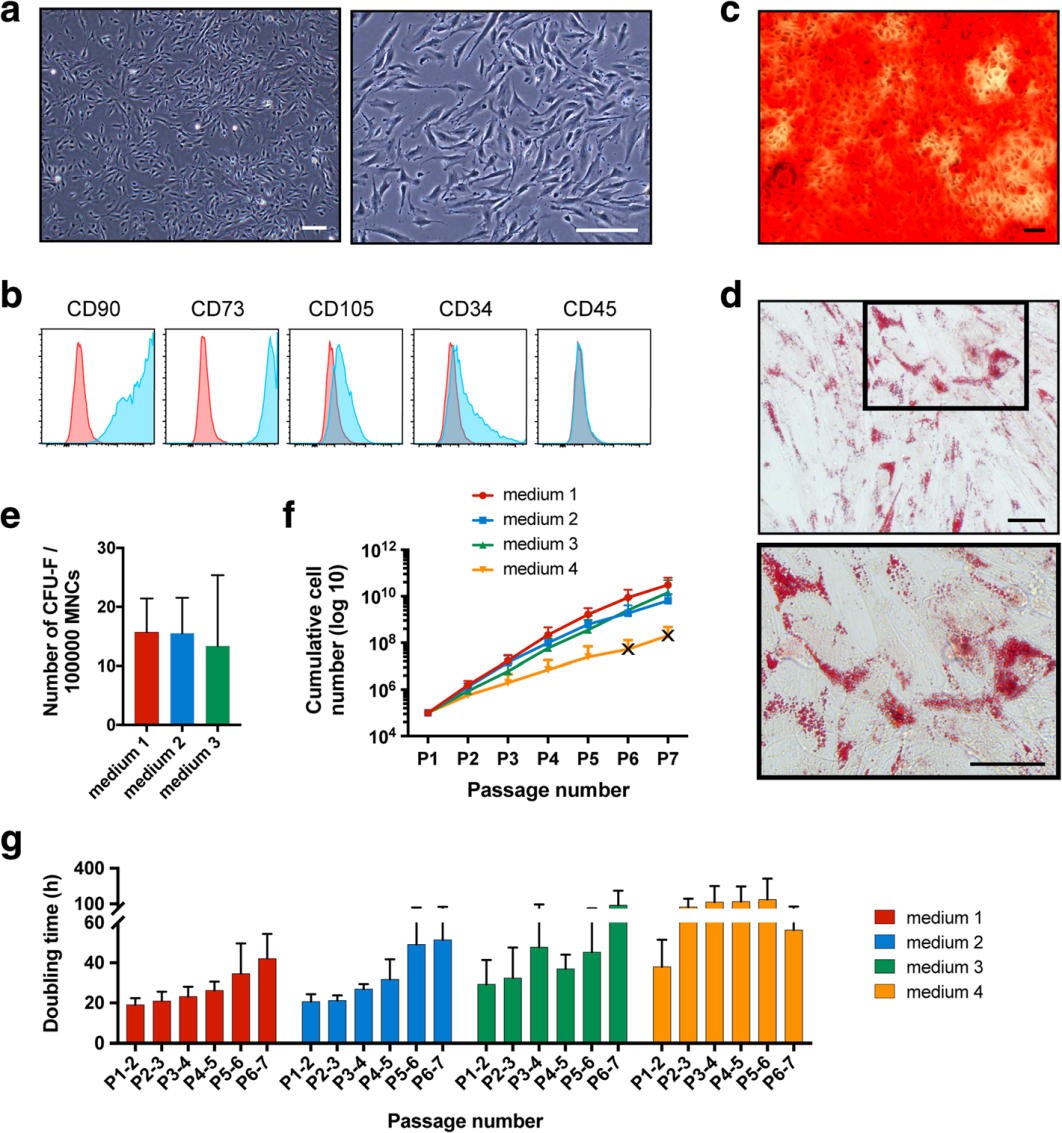
Highly proliferative cells in term amniotic fluid are MSCs with osteogenic and adipogenic differentiation capacity. **a** Representative microscopy images showing morphology of fibroblastic-like cell colonies. *Scale bars*: 100 μm. **b** Representative immunophenotypic FACS analyses of cells for MSC and non-MSC surface markers (*blue histograms*). Isotype controls represented (*red histograms*). **c** In-vitro osteogenic differentiation capacity of fibroblastic-like cells visualized by Alizarin Red staining to detect mineralized calcium secreted by osteoblasts. *Scale bar*: 100 μm. **d** Adipocyte differentiation visualized by Oil Red-O staining to detect lipid vacuoles of adipocytes. *Scale bars*: 50 μm. **e** CFU-F count per 100,000 mononuclear cells plated in media 1, 2, and 3, from five or six donor samples. **f, g** Growth curve and population doubling time (PDT, measured in hours) of seven MSC sample donors determined for seven passages in four different media. Cells were split every third day and counted at the end of every passage. TAF-MSCs showed a high proliferative activity. Data shown as mean ± SD. *X* signifies the termination of a sample in the study, due to a proliferation rate of less than 1 over 3 days of cultivation (this occurred in medium 4 only). *CFU-F* colony forming unit-fibroblasts

To identify cell types present in term amniotic fluid and assess their proliferation capacities, freshly isolated MNCs from eight samples were randomly chosen for plating in one, two, or all three culture media: media 1, 2, and 3. Following 11–14 days of culture, the formation of both spindle-shaped fibroblast-like cell colonies (CFU-F), and round-shaped epithelial-like cell colonies was observed (Additional file 2). The frequency of CFU-F was approximately 15 per 100,000 MNCs in each medium tested: medium 1, 15 ± 5 per 100,000 MNCs (*n* = 6); medium 2, 15 ± 6 per 100,000 MNCs (*n* = 5); and medium 3, 13 ± 12 (*n* = 6) per 100,000 MNCs (Fig. 2e). Clonal expansion of epithelioid-like cells showed a very slow proliferation rate which ultimately nulled after 25 days of cultivation. However, the fibroblastic-like cells were highly proliferative and capable of long-term in-vitro expansion (Fig. 2f, g). Upon extended culture of non-clonally derived cells, only the fibroblast-like cell type was seen in all cultures (similar to a clonally derived sample) suggesting a growth advantage of these cells over other cells initially present in the culture. Both the epithelioid and fibroblastic-like cell colonies were initially seen in all media tested. Expansion of epithelioid cells was also assessed in a media specific for epithelial cell growth (SAGM), where we also observed epithelial colony growth for a maximum of 3 weeks (data not shown).

### Adherent fibroblast-like cells in term amniotic fluid exhibit properties of MSCs

Following in-vitro expansion of fibroblast-like adherent cells cultured in media 1 and 2 from two randomly chosen samples, cells were then analysed by flow cytometry analysis for a panel of cell identity surface markers. As expected, they lacked expression of the pan-haematopoietic marker CD45, endothelial marker CD31, and a significant fraction of the cells were negative for CD34 (Fig. 2b and Additional file 3b). The cells were bright for MSC markers CD90 and CD73, and expressed CD105 at low levels (Fig. 2b). The low level of CD105 expression compared with MSCs isolated from other sources has been reported previously in studies of second and third-trimester amniotic fluid-derived MSCs [36, 37]. We also examined the expression of the pluripotency factor, *OCT4*, on both cultured and non-cultured freshly isolated cells, and observed undetectable levels of OCT4 expression on the cultured cells despite its evident expression in more than 90% of the freshly isolated cells (Additional file 3a,b).

Given the observed similarity in morphologies and cell surface marker expression of the cultured TAF-derived cells to MSCs, we set out to examine their differentiation capacity towards two of the definitive lineages of MSCs, osteoblasts, and adipocytes. Differentiation analyses confirmed the bipotency of these cells by detection of calcium deposits following Alizarin red staining, and lipid vacuole accumulation following Oil Red-O staining (Fig. 2c, d). Differentiation experiments were performed on three samples at passage 4 (including one clonally derived sample) expanded in media 1, 2, and 3. Taken together, these results demonstrate that the cells expanded in the various FCS-based media from term amniotic fluid are highly proliferative adherent progenitors with properties similar to those of MSCs (hereafter called TAF-MSCs).

### Term amniotic fluid-derived MSCs showed high proliferative activity

To study the growth characteristic and morphology of TAF-MSCs in various media, cells of seven independent donor samples were isolated and cultured for seven passages (29–38 days of cultivation including the time for initial colony formation) in the four FCS-based media. This revealed that the different media types influenced the cell growth kinetics, but had no significant effect on the morphology of the MSCs (Fig. 2f, g and Additional file 4). The average population doubling time (PDT) of MSCs during the first four passages in media 1, 2, 3, and 4 was 21 h ± 1.1 h, 22.9 h ±1.9 h, 36.4 h ± 5.7 h, and 75.4 h ± 22.1 h, respectively. The significant short PDT of TAF-MSCs indicates their high proliferative activity as a neonatal source of MSCs. We observed a gradual but insignificant increase in the PDT of the MSCs over time. In order to further study the effects of prolonged in-vitro expansion on proliferation rate and morphology of the MSCs in different media, three of the samples were continued to be cultured for longer time (10 passages, 41–46 days of cultivation including the time for initial colony formation). The results showed that the most pronounced changes in proliferation rate of the MSCs occur after a certain number of passages (Additional file 4a,b). The decrease in the proliferation rate of the MSCs was accompanied by some cells increasing in size suggestive of replicative senescence (Additional file 4c). Following cell expansion over several passages in media 4, some samples (two of the seven tested) showed slowing growth and did not reach passage 7. The cultures were therefore not continued, as indicated by crosses in cumulative cell number graphs (Fig. 2f and Additional file 4a).

### TAF-MSCs resemble MSCs isolated from a variety of sources

**Fig. 3:**
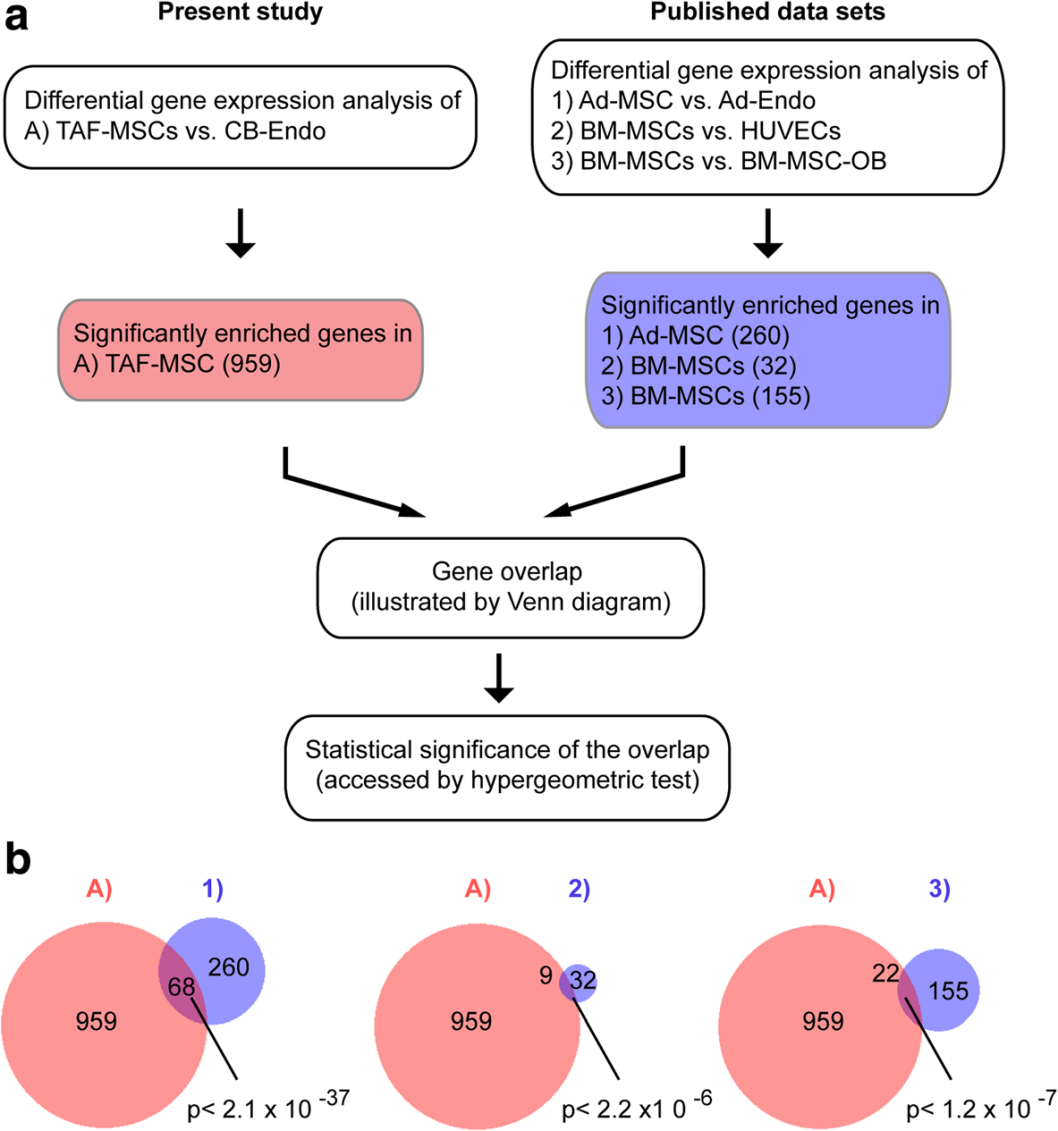
TAF-MSCs show a similar expression profile compared with expanded MSCs derived from other tissues. **a** Schematic diagram showing the main steps of analysis for assessing the transcriptional similarities of enriched genes in TAF-MSCs and MSCs from adult sources. **b** Venn diagrams illustrate significant similarities in the gene expression profiles of TAF-MSCs, BM-MSCs, and AD-MSCs. *Pink circles* total number of significantly differentially expressed genes between TAF-MSCs and CB-Endo (≥1.5 fc and *p* < 0.05); *blue circles* total number of significantly differentially expressed genes between AD-MSCs and AD-Endo (*left diagram*), between BM-MSCs and HUVECs (*middle diagram*), or between BM-MSCs and BM-MSC-OB (*right diagram*). *p* value calculated by hypergeometric statistical test indicates the significance of the overlap between each analysis. *AD-Endo* adipose tissue-derived endothelial cell, *AD-MSC* adipose tissue-derived mesenchymal stromal cell, *BM-MSC* bone marrow-derived mesenchymal stromal cell, *BM-MSC-OB* BM-MSCs induced to differentiate to osteoblasts, *CB-Endo* cord blood-derived endothelial cell, *HUVEC* human umbilical vein endothelial cell, *TAF-MSC* term amniotic fluid-derived mesenchymal stromal cell

In order to assess transcriptional similarities or differences between MSCs derived from term amniotic fluid and other sources (bone marrow (BM) and adipose tissue (AD)), comparative analyses of genes enriched in TAF-MSCs, BM-MSCs, and AD-MSCs were performed. To identify the functionally relevant, non-homeostasis-related genes enriched in TAF-MSCs, differential gene expression analyses between TAF-MSCs and a non-MSC (cord blood-derived endothelial cells (CB-Endo)) were performed (Additional file 5). Similarly, published studies that compared BM-MSCs and AD-MSCs with non-MSC endothelial cells (HUVECs or AD-Endo) or BM-MSC-derived osteoblast cells (BM-MSC-OB cells) [38, 39] were used to defined the genes that were significantly enriched in MSCs from those tissues. Comparison of genes enriched in TAF-MSCs with those enriched in AD-MSCs and BM-MSCs showed a highly significant overlap based on hypergeometric *p*-value statistical analysis (Fig. 3). These data indicate that TAF-MSCs share a gene expression profile significantly similar to MSCs derived from other tissues.

### TAF-MSCs can be reprogrammed to pluripotent stem cell state

Cellular reprogramming of TAF-MSCs could offer an expanded utility, for various medical applications. Thus, the reprogramming ability of TAF-MSCs to pluripotent stem cells was assessed by overexpressing the reprogramming factors *OCT4*, *SOX2*, *KLF4*, *C-MYC*, and *Lin28*. In total, nine iPS cell lines were generated from TAF-MSCs (TAF-iPS), which all showed similar levels of *OCT4* and *Nanog* pluripotency gene expression as compared with human embryonic stem (hES) cell lines, and had the potential to form teratomas (Additional file 6). Additional iPS cell lines were also generated from human umbilical cord blood endothelial cells (CB-iPS) using the same reprogramming protocol. The CB-iPS cell lines were used for comparison in subsequent differentiation analyses of TAF-iPS cell lines along with multiple hES cell lines.

### Pluripotent stem cell lines derived from TAF-MSCs efficiently differentiate into haematopoietic and neural cell lineages

**Fig. 4:**
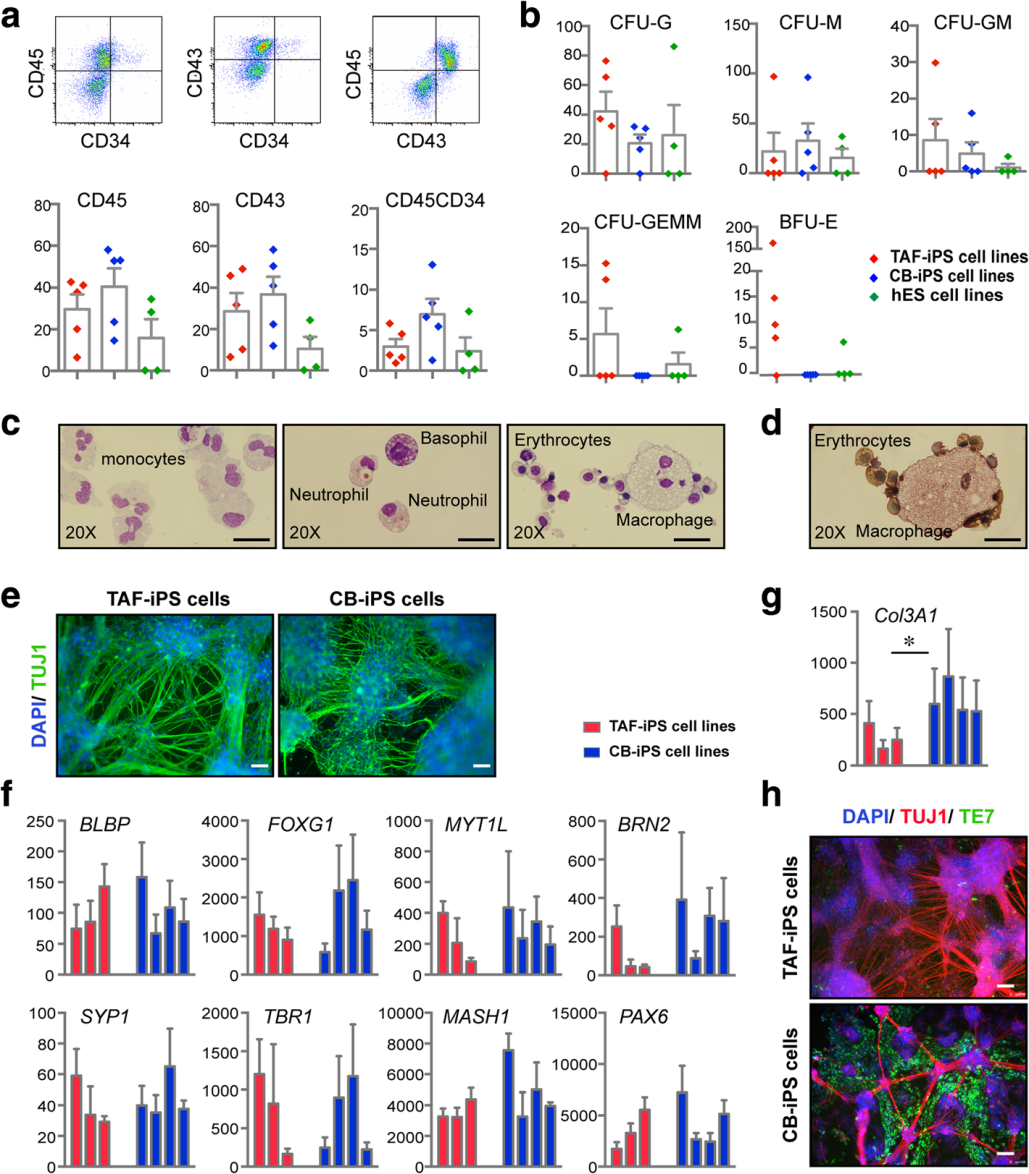
TAF-iPS cell lines show efficient differentiation capacity into haematopoietic and neural cell lineages. **a** Representative FACS plots and percentage of haematopoietic cell outputs following differentiation of five TAF-iPS cell lines, five CB-iPS cell lines, and four hES cell lines (HUES-3, HUES-6, H1, H9). Each dot represents a distinct pluripotent stem cell line. **b** Colony forming unit (CFU) counts per 10,000 plated cells. *CFU-G* granulocyte, *CFU-M* macrophage, *CFU-GM* granulocyte/macrophage, *CFU-GEMM* granulocyte/erythroid/macrophage, *BFU-E* burst forming unit—erythroid. **c** Representative images from cytospin preparation of CFU-G, CFU-M, and BFU-E stained with May–Grünwald–Giemsa. **d** Benzidine staining demonstrating haemoglobinized erythroid cells. **e** Representative immunofluorescent images of TUJ1 staining in TAF-iPS and CB-iPS cell cultures after neural induction. **f, g** Quantitative gene expression analyses of eight neural markers and non-neural marker *COL3A1* after neural induction. Data represent an average of three to five independent experiments for each cell line and three technical replicates per sample, calculated as fold change expression relative to undifferentiated hES cell line, H9. **h** Immunofluorescent staining of TAF-iPS and CB-iPS cell lines after neural induction for TUJ1 and the non-neuronal marker TE7. Data represented as mean ± SEM. Statistical analysis was performed using Student’s *t* test. *p < 0.05. *Scale bars*: all 100 μm. *CB-iPS* *cell* cord blood-derived induced pluripotent stem cell, *hES* *cell* human embryonic stem cell, *TAF-iPS* *cell* term amniotic fluid-derived induced pluripotent stem cell

In order to evaluate the differentiation potential of TAF-iPS cell lines across multiple germ layers, five randomly selected TAF-iPS lines were differentiated towards haematopoietic cell and neuronal lineages (Fig. 4). Firstly, haematopoietic lineage differentiation potential was assessed for the TAF-iPS lines and compared with control pluripotent stem cell lines (CB-iPS and hES cell lines) using FACS and haematopoietic progenitor CFU assays. TAF-iPS cell lines could generate comparable numbers of haematopoietic cells and functional haematopoietic progenitors as the pluripotent control lines (Fig. 4a, b). However, TAF-iPS cell lines showed a significantly higher efficiency in erythroid lineage differentiation as assessed by BFU-E formation compared with the control lines. The normal morphological characteristics of monocytes, granulocytes, and erythroid cells of CFU-M, CFU-G, and BFU-E colonies were confirmed by May–Grünwald–Giemsa staining (Fig. 4c). In addition, hemoglobinization of erythroid cells was confirmed by benzidine staining (Fig. 4d). The lymphoid differentiation potential of the TAF-iPS cells was confirmed by the ability to develop phenotypic NK (CD16/CD56-positive cells) and mature T cells (CD4/CD3 double-positive cells) (Additional file 7). These data show that the TAF-iPS cell lines possess efficient differentiation potential towards all haematopoietic cell lineages tested.

Next, the neural (ectoderm) differentiation potential of the TAF-iPS cell lines was assessed. Following differentiation, immunofluorescent analyses confirmed the generation of a high number of TUJ1^+^ neural cells from both TAF-iPS and CB-iPS cell cultures (Fig. 4e). We then assessed the expression levels of eight pan or specific neural lineage genes, and a non-neural marker *COL3A1* (Fig. 4f, g). No statistically significant differences in the expression level of neural cell marker between TAF-iPS and CB-iPS cell cultures were observed (Fig. 4f). However, *COL3A1* was expressed at a significantly lower level in TAF-iPS cell cultures compared with CB-iPS cell cultures (Fig. 4g). Immunofluorescent staining of an additional non-neural marker TE-7, confirmed this observation, suggesting that neural-induced TAF-iPS cell cultures contain less non-neural cells and are more homogeneous compared with neural-induced CB-iPS cell cultures (Fig. 4h). Collectively, haematopoietic and neural differentiation analyses show that TAF-iPS cells possess broad differentiation potential towards both mesodermal and ectodermal cell lineages.

## Discussion

This study showed that term amniotic fluid is a relatively rich source of MSCs having high expansion capacities and features similar to MSCs isolated from other sources. We also showed that harvesting amniotic fluid at term caesarean section deliveries by catheter resulted in much higher collection volumes compared with 10–20 ml volumes that were harvested in previous studies of term or pre-term amniotic fluid [24–26]. We were able to harvest on average about 400 ml of fluid from the 20 samples. Comparing the TAF-MSCs described in this study with MSCs isolated from BM and AD, we show that they share a significantly similar transcriptional profile. Taken together, these results suggest that TAF-MSCs may be an alternative and complimentary source of MSCs with features making them highly attractive for further investigation in in-vivo pre-clinical functional assessments.

There are only a limited number of publications characterizing term human amniotic fluid cells [24–26]. In line with these previous studies of term amniotic fluid and studies of second-trimester amniotic fluid [40], we have identified cells with either spindle-shaped or round-shaped morphology from term amniotic fluid, and assessed their growth characteristics. We have shown that the highly proliferative cells present in term amniotic fluid have the properties of MSCs, as characterized by cell surface markers, differentiation potential, and gene expression analysis. We also demonstrated the high proliferation capacity of TAF-MSCs. The three donor samples that were cultured for a longer period of time (10 passages, 41–46 days of cultivation including the time for initial colony formation) reached a maximum cumulative cell number of 10^13^ (range 10^9^–10^13^, depending on the media type). The proliferation rate of TAF-MSCs is greater than adult sources of MSCs [21, 22], with the fastest doubling rate seen in this study being approximately 14 hours. We have observed differences in proliferation rates of cells growing in different media, which, as previously described for other MSC sources, could be due to different growth factors that are present in the respective media [41]. We have also observed a slow decrease in proliferation rate during the earlier passages of MSC expansion in all the media. A more rapid decrease in the proliferation rate of MSCs was seen only at higher passages (the actual passage number differs for each media; Additional file 4a,b). MSCs also demonstrated a continuous increase in fraction of cells enlarging in size over time in accordance with previous reports of MSCs from other sources [17, 41]. In addition, reduced differentiation and therapeutic potential of MSCs has been associated with prolonged in-vitro culture [16–19]. Therefore, prolonged in-vitro cultures are not recommended, and in order to obtain the number of cells necessary for clinical applications, using the most abundant MSC sources that allow for short-term in-vitro expansion is advantageous. However, the MSC frequency is not the sole factor that needs to be considered when selecting the most appropriate MSC source for clinical application. Differences in in-vivo developmental potential of MSCs from distinct sources are also of major importance for consideration to ensure the efficacy and safety of the therapy [6, 7]. Thus, each MSC source needs to be evaluated for its in-vivo developmental potential and assure that these properties match the required criteria of each specific clinical application.

Reprogramming of cells from TAF-MSCs to true pluripotent states may offer an expanded utility of these cells for disease modelling, drug discovery and testing, and regenerative medicine. As a neonatal source, TAF-MSCs represent a valuable starting cell material for iPS cell generation considering their reduced exposure to mutagens compared with adult sources and their accessibility as a currently discarded material. Moreover, their adherent highly proliferative nature is also amenable to current reprogramming technologies. We demonstrated that TAF-MSCs can be reprogrammed to generate induced pluripotent stem (iPS) cells, which can then be efficiently differentiated to both haematopoietic and neural lineages. The TAF-iPS cell lines generated in this study showed significantly higher differentiation efficiency towards the erythroid lineage. It would be interesting to generate TAF-iPS cells from additional donors and assess whether efficient differentiation towards erythroid lineages is a general feature of TAF-iPS cell lines, or if it is a donor-related issue [42]. TAF-iPS cells could also differentiate into phenotypic NK (CD16/CD56-positive cells) and mature T cells (CD4/CD3 double-positive cells). The generation of mature lymphoid cells from pluripotent stem cell sources has been inefficient and remains a challenge [43]. When neural differentiation was induced, TAF-iPS cell lines demonstrated comparable neural differentiation ability in comparison with CB-iPS cell lines, and significantly less mesodermal differentiation as measured by *COL3A1*, thus generating a more homogeneous culture. The broad potential donor base of these cells and their neonatal origin support the idea of establishing iPS cell bank of normal and diseases patient samples from these cells. Together, our data show that TAF-MSCs have features desirable for generating iPS cells, and therefore potentially have value in pluripotent stem cell-based regenerative medicine applications.

Several studies have reported that the majority of isolated cells from second and third-trimester amniotic fluid express the stem cell marker *OCT4* (*POU5F1*) [36, 44–47]. The expression of *OCT4* has been associated with pluripotency, and this was further advanced by showing the capacity of single or clonal *OCT4*-positive cells to differentiate towards various cell lineages [36, 45]. Comparative study of amniotic fluid-derived MSCs from second and third-trimester pregnancies revealed comparable levels of *OCT4* expression in these cells regardless of their gestational age [48]. Consistent with previous reports, we have also identified that the majority of freshly isolated cells from term amniotic fluid are *OCT4*-positive. Given the interest in further evaluating *OCT4*-positive amniotic fluid cells in pre-clinical studies, here we demonstrate that the closed catheter extraction system is compatible for extraction of large number of *OCT4*-positive cells. We and others have shown that *OCT4* expression is lost over time in culture [46], but it has been reported that hypoxia helps maintaining *OCT4* expression and stem cell properties [49]. The closed catheter-based system can minimize air exposure compared with active suction devices.

The average number of caesarean section deliveries in OECD countries is approximately 3 million per year (approximately 26% of all births) (Statlink: http://dx.doi.org/10.1787/888932524887). Depending on the exclusion criteria for the procedure of donating term amniotic fluid (e.g. emergency C-section), and given that not every eligible donor would probably accept to participate, the actual numbers of term amniotic fluid samples collectable is difficult to predict. However, unlike collection of MSCs from some traditional sources where age-related concerns for cellular function and age/health restrictions for the medical extraction procedure exist, term amniotic fluid acquisition during a planned CS delivery could potentially provide relatively large numbers of MSCs without these complicating factors.

## Conclusions

In this study, we have demonstrated that retrieval of significant volumes of term amniotic fluid is feasible in connection with planned caesarean sections, allowing for access to an unexploited reserve of MSCs by the use of a novel collection system which maintains sterility, minimizes air exposure, and reduces fluid-shear forces on the collected material. TAF-MSCs were the most proliferative adherent cells in the fluid in the media tested and showed similar characteristics to MSCs isolated from traditional sources. These cells were reprogrammed to pluripotent stem cell state and were able to efficiently differentiate to haematopoietic and neural cell lineages. The broad potential donor base of amniotic fluid material, and high cell yields of the procedure, make this currently untapped source of cells attractive for further evaluation in bio-banking, cell therapy, disease modelling, and regenerative medicine applications.

## Additional files

Additional file 1: is an extended description of the experimental procedure [50]. (DOC 42 kb)

Additional file 2: Showing representative microscopy images of epithelioid-like (**a**) and fibroblastic-like (**b**) cell colonies which were formed after plating of the term amniotic fluid mononuclear cells. *Scale bars*: 100 μm. (TIF 5721 kb)

Additional file 3: Showing (**a**) endogenous expression of the pluripotency marker *OCT4* in freshly isolated term amniotic fluid cells (*left panel*) and culture-expanded term amniotic fluid-derived cells (*right panel*). (**b**) OCT4 expression and cell surface expression of CD45, CD34, and CD31 in freshly isolated term amniotic fluid cells (*upper panel*) and culture-expanded (*lower panel*) term amniotic fluid-derived cells. (TIF 3593 kb)

Additional file 4: Showing long-term in-vitro culture of TAF-MSCs. (**a**, **b**) Long-term growth curve and population doubling time (PDT, measured in hours) are demonstrated for three MSC sample donors cultivated in four different media. These data demonstrate the effect of prolonged in-vitro culture on proliferation capacity of MSCs. Data shown as mean ± SD. *X* signifies the termination of a sample in the study, due to a proliferation rate of less than 1 over 3 days of cultivation. (**c**) Representative microscopy images demonstrate the changes in morphological size of MSCs with increasing passages in four different media. *Scale bars*: 100 μm. (TIF 17404 kb)

Additional file 5: Showing the list of differentially expressed genes between TAF-MSCs and CB-Endo cells. *TAF-MSCs* term amniotic fluid-derived MSCs, *CB-Endo cells* cord blood-derived endothelial cells. (XLSX 4454 kb)

Additional file 6: Showing TAF-iPS cell lines are pluripotent. (**a**) Q-RT-PCR analyses of *OCT4*, *SOX2*, *KLF4*, *C*-*MYC*, *NANOG*, *DNMT3B*, *TDGF1 (CRIPTO)*, *and ZFP42 (REX1)* expression in nine TAF-iPS, CB-iPS and hES cell lines. Samples were normalized against the internal control (*GAPDH*) and plotted (log_10_ scale) relative to the expression level in the hES cell line HUES-3, which is arbitrarily set to a value of 1. (**b**) Histological analyses of teratomas generated by TAF-iPS cell lines reveal the presence of tissues with characteristic structures of all three germ layers. Representative images of haematoxylin and eosin (H&E)-stained teratoma sections, generated from a TAF-iPS cell line following subcutaneous injection of 500,000 iPS cells into NSG mice. (TIF 14420 kb)

Additional file 7: Showing TAF-iPS are capable of differentiating towards lymphoid lineages. (**a**) Representative flow cytometry plots showing the phonotype of T cells generated from 100 sorted CD43^+^CD34^+^ cells after in-vitro co-culture on OP9-DL1 stroma for 4 weeks. CD33 and Topro were used for exclusion of myeloid cells and GFP-expressing OP9 and OP9-DL1 cells from analyses, respectively. (**b**) Representative plots showing the phonotype of NK cells generated from 100 sorted CD43^+^CD34^+^ cells after in-vitro co-culture on OP9 stroma for 4 weeks. (TIF 6590 kb)

## Abbreviations

AD-Endo: Adipose tissue-derived endothelial cell
AD-MSC: Adipose tissue-derived mesenchymal stromal cell
BM-MSC: Bone marrow-derived mesenchymal stromal cell
BM-MSC-OB: BM-MSCs induced to differentiate to osteoblasts
CB-Endo: Cord blood-derived endothelial cell
CB-iPS cell: Cord blood-derived induced pluripotent stem cell
CFU assay: Colony forming unit assay
CS: Caesarean section
EB: Embryoid body
HUVEC: Human umbilical vein endothelial cell
iPS cell: Induced pluripotent stem cell
MSC: Mesenchymal stromal cell
TAF: Term amniotic fluid
TAF-iPS: Term amniotic fluid-derived induced pluripotent stem
TAF-MSC: Term amniotic fluid-derived mesenchymal stromal cell
