# Supplementary material for: Term amniotic fluid: an unexploited reserve of mesenchymal stromal cells for reprogramming and potential cell therapy applications
Source: Stem Cell Res Ther. 2017 Aug 25;8:190. doi: 10.1186/s13287-017-0582-6 (PMC5574087; doi:10.1186/s13287-017-0582-6)
Supplement: Supplementary file 1 — is an extended description of the experimental procedure [50]. (DOC 42 kb) [file 13287_2017_582_MOESM1_ESM.doc]

**Extended description of experimental procedure**

**TAF-MSC reprogramming to pluripotent stem cell**

To generate iPS cell lines, we used a tet-inducible expression system. TAF-MSCs were transduced by lentiviral vectors (FU-tet-o-h*OCT4*, -h*SOX2*, -h*KLF4*, -h*CMYC*, -h*LIN28*, -*GFP*, FUdeltaGW-*rtTA)* to achieve 80% transduction efficiency. Once reaching 80% transduction efficiency, cells were individualized by trypsin and plated on murine embryonic fibroblast feeder cells in respective media supplemented by 1μg/mL Dox. 48h and 72h post-plating, the culture medium was changed to 50% and 100% hESC medium supplemented by 1μg/mL Dox, respectively. The hES cell medium supplemented by 1μg/mL Dox was changed every second day until iPS-like colonies appeared. The iPS-like colonies (with sharp borders and packed cells with large nuclei) were picked at around day 20 to day 30 after viral transduction.

**Pluripotency analyses of generated iPS cell lines**

Chromosomal studies of the iPS cell lines were performed using standard protocols for high resolution G-banding. For *in-vivo* teratoma analyses, severe combined immunodeficient (Nod/Scid/Il2rg^-/-^) mice obtained from Jackson Laboratories were anesthetized and subcutaneously injected with approximately 7-10 million TAF-iPS cells resuspended in 50 μl of PBS +5% FCS. Mice were euthanized when the tumor reached a visual large and palpable size. The tumors were fixed in 4% Paraformaldehyde, Paraffin sectioned and stained using hematoxylin and eosin (H&E). For Q-RT-PCR analyses of pluripotency markers, total messenger RNA was prepared using Trizol reagent (Invitrogen) and phenol-chloroform extraction method as described by Invitrogen Life Technologies protocol. DNase I (Qiagene) was used to remove any residual genomic DNA. 1μg of RNA was used to synthesize cDNA using the SuperScript III Reverse Transcriptase Kit (Invitrogen). 0.1 μl of the final reaction was used to quantify gene expression by qPCR using the SYBR® Green I, and the qPCR Supermix-UDG with Rox (Invitrogen). Quantitative Real-Time was performed on a 7900HT Fast Real-Time PCR system (Applied Biosystems). Values of gene expression were normalized using GAPDH expression and are shown as fold changes relative to the value of the sample control. All the samples were done by duplicate using primers as described by Brennand KJ. et al [50]. Size of amplicons and absence of nonspecific products were controlled by melting curves.

**Lymphoid differentiation analyses**

For lymphoid differentiation analysis, one hundred sorted CD43CD34 double positive cells were plated on approximately 80% confluent OP9 or OP9-DL1 monolayer stroma cells in OPTI-MEM medium (Invitrogen) supplemented by 10% FCS (Sigma-Aldrich), 1% Penicillin /Streptomycin (Sigma-Aldrich), 1% L-GLu, 0,1 mM 2-β-mercaptoethanol (Sigma-Aldrich) and cytokines combinations of IL-3 (only the first week, 5ng/mL), IL-7 (5ng/mL, only the first week of OP9-DL1 culture and the entire period of OP9 cultures), c-KIT ligand (SCF) (10ng/mL), Flt3 ligand (FLT3L) (10ng/mL), IL-15 (10 ng/mL) and IL2 (5ng/mL, was added only to OP9-DL1 culture). All cytokines were purchased from PeproTech. OP9 and OP9-DL1 GFP-expressing stroma cells were kindly provided by Dr. J.C. Zúñiga-Pflücker, and cultures were carried out as previously described [34]. Cells were cultured at 37°C for 4 weeks on OP9 and OP9-DL1, with weekly changes of the half of the medium. Following 4 weeks of differentiation, cells were harvested and assayed for T and NK cell surface marker expression. CD33 was used for exclusion of myeloid cells and TO-PRO-1 was used to exclude dead cells and GFP-expressing OP9 and OP9-DL1 stroma cells from analyses. Wells with only stroma cells were used as negative control. Wells were considered as positive when at least 30 viable human cells were detected and were scored positive for mature blood lineages when at least 20 cells with the required phenotype were detected.

**Neural differentiation of iPS cell lines**

Neural induction was stimulated by embryoid body formation as previously described in [35], but in the absence of SB431542 and Noggin from the medium. On day 4, embryoid bodies were attached onto coated plates, and resulting neural colonies were passaged on day 11 and matured into neurons for 10 additional days in medium containing BDNF (20 ng/ml), cAMP (0.5 mM), Ascorbic acid (200 uM), GDNF (10 ng/ml) and DAPT (1 uM).

**qRT-PCR analyses**

For qRT-PCR analyses, RNA was isolated from cell cultures using the RNeasy Micro kit (QIAGEN). cDNA synthesis was then performed using up to 1 μg of RNA from each sample, random hexamer primers, and Superscript III enzyme (Invitrogen). The cDNA was amplified and detected by SYBR Green Master Mix (Roche) on the LightCycler 480 instrument using a 2-step protocol. All quantitative RT-PCR (qRT-PCR) samples were run in technical triplicates, and the average Ct-values were used for calculations. Data were first normalized to ß-actin or GAPDH housekeeping gene, and fold changes were calculated relative to undifferentiated hES cell line (H9).
