# Supplementary figures and images for: Term amniotic fluid: an unexploited reserve of mesenchymal stromal cells for reprogramming and potential cell therapy applications
Source: Stem Cell Res Ther. 2017 Aug 25;8:190. doi: 10.1186/s13287-017-0582-6 (PMC5574087; doi:10.1186/s13287-017-0582-6)

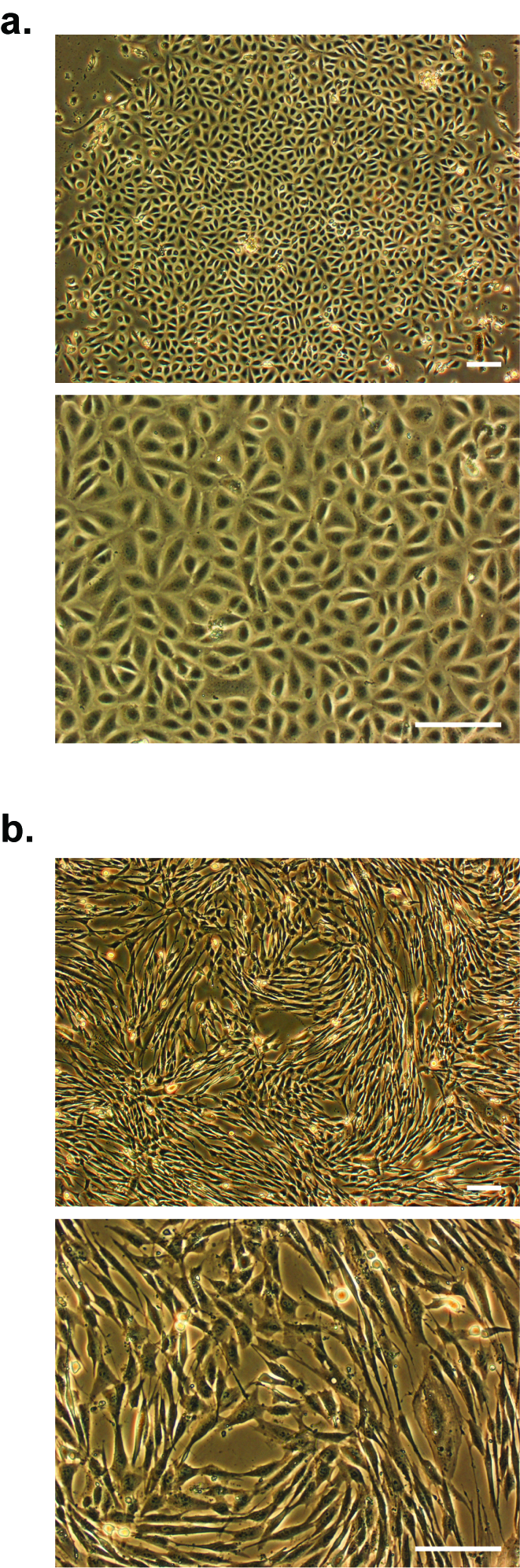

Supplement: Supplementary file 2 — Showing representative microscopy images of epithelioid-like (a) and fibroblastic-like (b) cell colonies which were formed after plating of the term amniotic fluid mononuclear cells. Scale bars: 100 μm. (TIF 5721 kb) [file 13287_2017_582_MOESM2_ESM.tif]

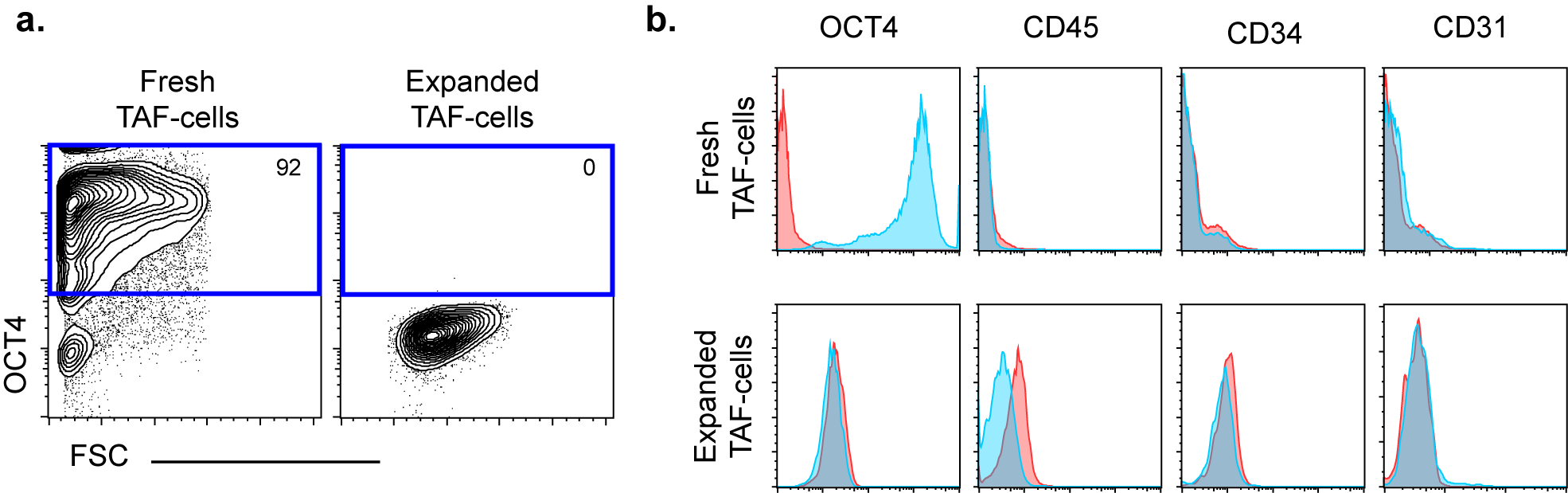

Supplement: Supplementary file 3 — Showing (a) endogenous expression of the pluripotency marker OCT4 in freshly isolated term amniotic fluid cells (left panel) and culture-expanded term amniotic fluid-derived cells (right panel). (b) OCT4 expression and cell surface expression of CD45, CD34, and CD31 in freshly isolated term amniotic fluid cells (upper panel) and culture-expanded (lower panel) term amniotic fluid-derived cells. (TIF 3593 kb) [file 13287_2017_582_MOESM3_ESM.tif]

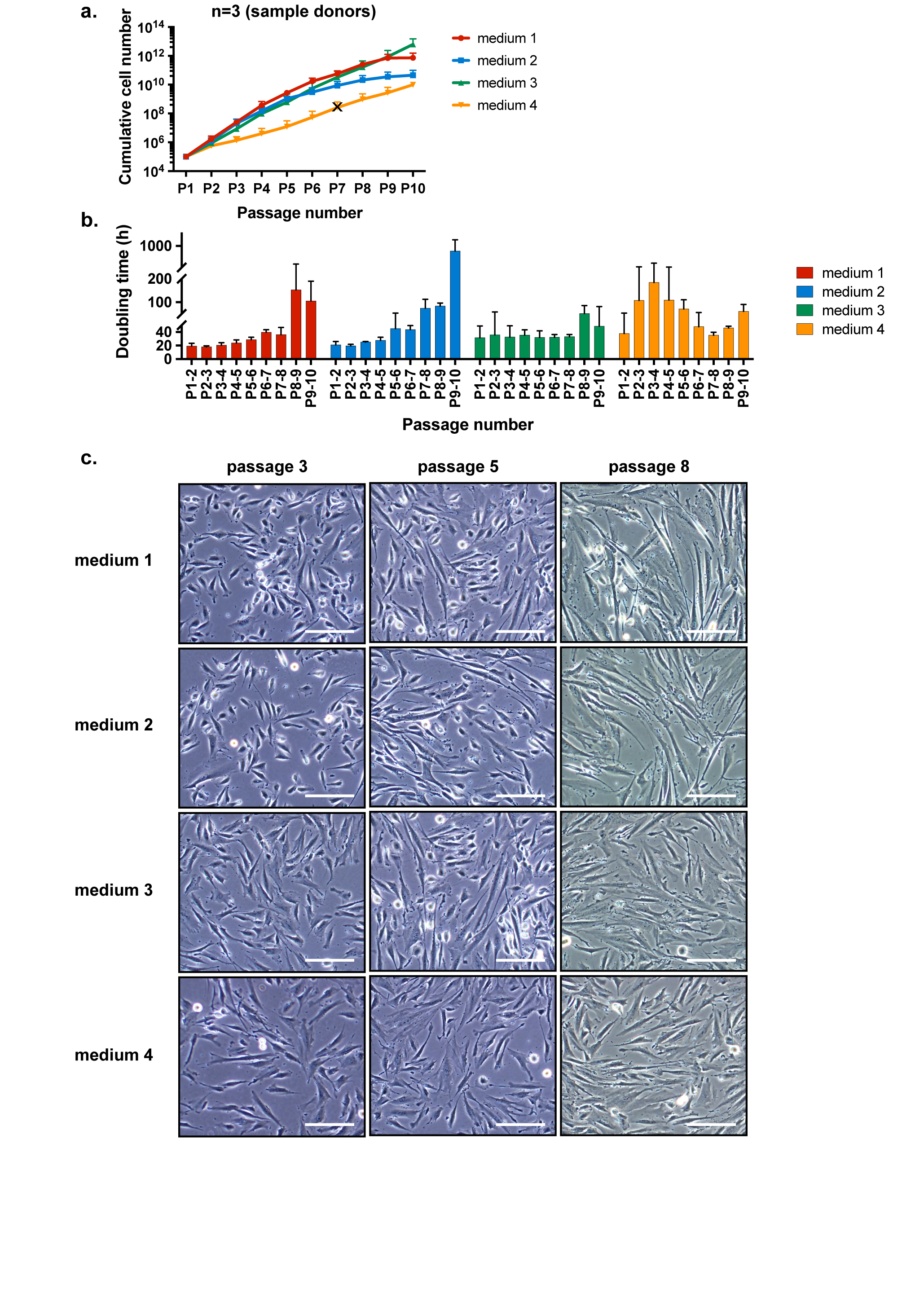

Supplement: Supplementary file 4 — Showing long-term in-vitro culture of TAF-MSCs. (a, b) Long-term growth curve and population doubling time (PDT, measured in hours) are demonstrated for three MSC sample donors cultivated in four different media. These data demonstrate the effect of prolonged in-vitro culture on proliferation capacity of MSCs. Data shown as mean ± SD. X signifies the termination of a sample in the study, due to a proliferation rate of less than 1 over 3 days of cultivation. (c) Representative microscopy images demonstrate the changes in morphological size of MSCs with increasing passages in four different media. Scale bars: 100 μm. (TIF 17404 kb) [file 13287_2017_582_MOESM4_ESM.tif]

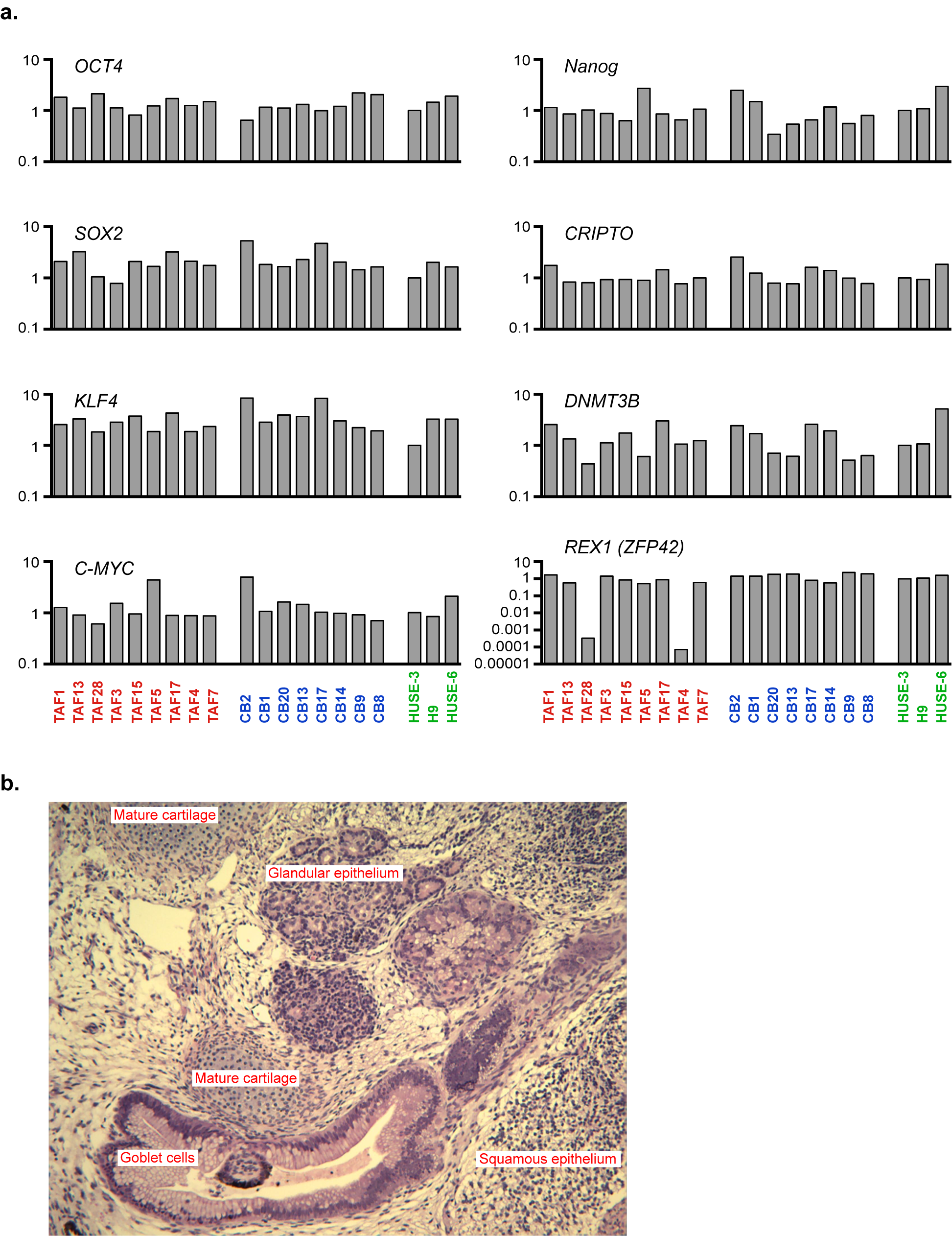

Supplement: Supplementary file 6 — Showing TAF-iPS cell lines are pluripotent. (a) Q-RT-PCR analyses of OCT4, SOX2, KLF4, C-MYC, NANOG, DNMT3B, TDGF1 (CRIPTO), and ZFP42 (REX1) expression in nine TAF-iPS, CB-iPS and hES cell lines. Samples were normalized against the internal control (GAPDH) and plotted (log10 scale) relative to the expression level in the hES cell line HUES-3, which is arbitrarily set to a value of 1. (b) Histological analyses of teratomas generated by TAF-iPS cell lines reveal the presence of tissues with characteristic structures of all three germ layers. Representative images of haematoxylin and eosin (H&E)-stained teratoma sections, generated from a TAF-iPS cell line following subcutaneous injection of 500,000 iPS cells into NSG mice. (TIF 14420 kb) [file 13287_2017_582_MOESM6_ESM.tif]

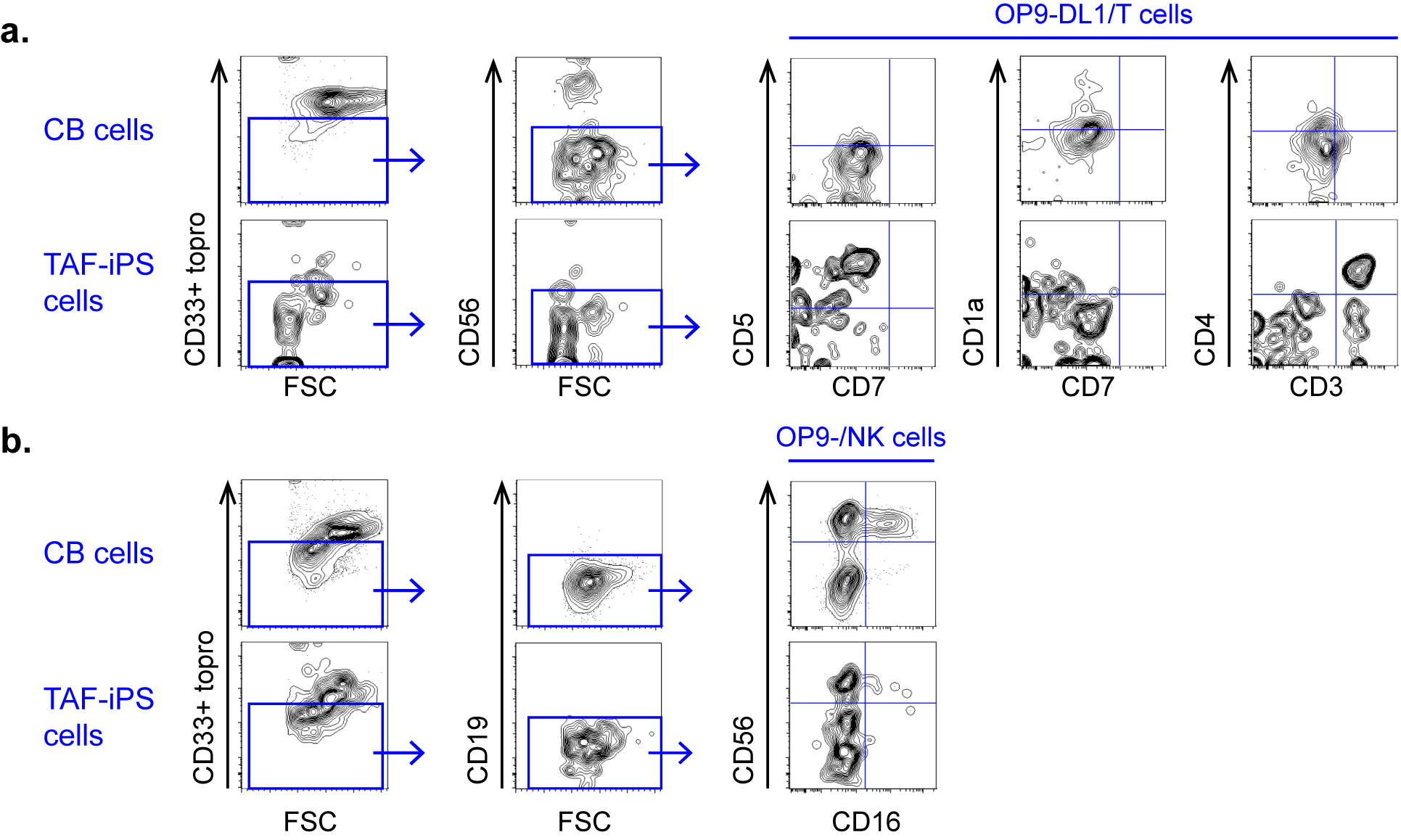

Supplement: Supplementary file 7 — Showing TAF-iPS are capable of differentiating towards lymphoid lineages. (a) Representative flow cytometry plots showing the phonotype of T cells generated from 100 sorted CD43+CD34+ cells after in-vitro co-culture on OP9-DL1 stroma for 4 weeks. CD33 and Topro were used for exclusion of myeloid cells and GFP-expressing OP9 and OP9-DL1 cells from analyses, respectively. (b) Representative plots showing the phonotype of NK cells generated from 100 sorted CD43+CD34+ cells after in-vitro co-culture on OP9 stroma for 4 weeks. (TIF 6590 kb) [file 13287_2017_582_MOESM7_ESM.tif]
